# High Sensitivity Optically Pumped Quantum Magnetometer

**DOI:** 10.1155/2013/858379

**Published:** 2013-05-26

**Authors:** Valentina Tiporlini, Kamal Alameh

**Affiliations:** Electron Science Research Institute, Edith Cowan University, 270 Joondalup Drive, Joondalup, WA 6027, Australia

## Abstract

Quantum magnetometers based on optical pumping can achieve sensitivity as high as what SQUID-based devices can attain. In this paper, we discuss the principle of operation and the optimal design of an optically pumped quantum magnetometer. The ultimate intrinsic sensitivity is calculated showing that optimal performance of the magnetometer is attained with an optical pump power of 20 **μ**W and an operation temperature of 48°C. Results show that the ultimate intrinsic sensitivity of the quantum magnetometer that can be achieved is 327 fT/Hz^1/2^ over a bandwidth of 26 Hz and that this sensitivity drops to 130 pT/Hz^1/2^ in the presence of environmental noise. The quantum magnetometer is shown to be capable of detecting a sinusoidal magnetic field of amplitude as low as 15 pT oscillating at 25 Hz.

## 1. Introduction

Optically pumped quantum magnetometers are based on the use of the atomic-spin-dependent optical properties of a medium. The general principle of operation of optically pumped quantum magnetometers is described in detail in [1]. A circularly polarized laser light transmitted through a glass cell containing a vapor of alkali atoms (e.g., Cs) resonates when its frequency equals the first absorption line of the alkali atoms, thus creating a spin alignment that precesses with a frequency proportional to the modulus of an externally applied magnetic field, *B*_0_ (Larmor frequency, *w*_*L*_ = *γ*|*B*_0_|). If this precession is coherently driven by an rf magnetic field, *B*_rf_ (oscillating at frequency *w*_rf_), the absorption coefficient of the alkali medium changes, thus modulating the transmitted optical intensity. By applying a very small oscillating magnetic field and maintaining the driving rf magnetic field resonant with the Larmor frequency, that is, *w*_rf_ = *w*_*L*_, the very small oscillating magnetic field can be determined. Several optical magnetometers have been developed, demonstrating sensitivities as high as those of superconducting quantum interference device- (SQUID-) based magnetometers. However, SQUID-based magnetometers must be operated at very low temperatures, thus requiring expensive cooling mechanisms. On the other hand, optical magnetometers not only work at room temperature but also have the potential of miniaturization, making them more practical for many applications [2, 3]. 

The basic operation mode of optical magnetometers involves the following mechanisms: Coherent Population Trapping (CPT), Nonlinear-Magneto Optical Rotation (NMOR), Spin-Exchange Relaxation Free (SERF) regime, and Mx magnetometer. The CPT method is an all-optical technique based on the use of two light sources (probe and pump) with orthogonal polarizations that create a coupling between two Zeeman sublevels located in different hyperfine states. When the modulation of the probe light is in resonance with the Zeeman splitting frequency ground sublevels a decrement in the light intensity can be observed. CPT-based magnetometers work in a scalar mode. The application of CPT-based magnetometers to cardiosignal detection has been demonstrated showing a sensitivity of 1 pT/Hz^1/2^ in an unshielded environment [4].

The NMOR technique is based on the linear magneto optical (Faraday) rotation principle. When the light modifies the properties of the medium, a nonlinear magneto optical rotation occurs. This happens when the light frequency is resonant with the atomic transitions of the medium, thus allowing the optical absorption to take place. The dynamic range of an NMOR-based magnetometer is limited by the width of the resonance, and the sensitivity that can be obtained is around 0.15 pT/Hz^1/2^ [5], which is adequate for geomagnetic measurements. The magnetometers operating in the SERF regime are also based on NMOR principle. However, such magnetometers have limited sensitivities due to depolarization caused by various atoms interaction types. The dominant type of these interactions is the spin-exchange collisions that can change the hyperfine state of the atoms while preserving the total angular momentum of the colliding atom pair. This results in a decoherent precession of the atom ensemble in the presence of a magnetic field, which makes the measurement of the Larmor frequency difficult. However, decoherence due to spin-exchange collisions can be completely eliminated if the spin-exchange collisions occur faster than the precession frequency of the atoms. This kind of optical magnetometers can reach a sensitivity of 0.54 fT/Hz^1/2^ [6]. The Mx magnetometers are so called because an rf oscillating magnetic field is supplied to the atoms to modulate the *x*-component of the magnetization vector inside the vapor cell. The phase difference between the driving rf signal and the probe light transmitted through the vapor cell gives a direct measurement of the Larmor frequency. This kind of magnetometer is vastly used in magnetocardiography, and it can reach sensitivity of 99 fT/Hz^1/2^ [7]. Optical magnetometers based on CPT and SERF regime operate in zero-magnetic-fieldconditions while the others require a weak magnetic field to induce Zeeman splitting. Between all the operation modes of optical magnetometers, the Mx configuration and SERF regime can achieve the highest sensitivity. An attractive feature of magnetometers working in the SERF regime is that they do not need an rf magnetic field or magnetic coils in the proximity of the vapor cell, which could create crosstalk problems in array configurations. On the other hand, the SERF regime requires higher temperature to attain high sensitivity and only works in very weak magnetic fields because the frequency of collisions must be higher than the Larmor frequency. This makes the SERF regime much more susceptible to environmental noise. 

In this paper, we describe the principle of operation of an Mx-configuration-based optically pumped quantum magnetometer and demonstrate experimentally the dependence of its sensitivity and bandwidth upon the light power and the alkali vapor temperature. The paper is organized as follows. In Section 2, we explain the principle of operation of an optical Mx magnetometer. In Section 3, the experimental setup is described. Finally in Section 4, we present and discuss the obtained experimental results.

## 2. Atomic Mx Magnetometer

**Figure 1:**
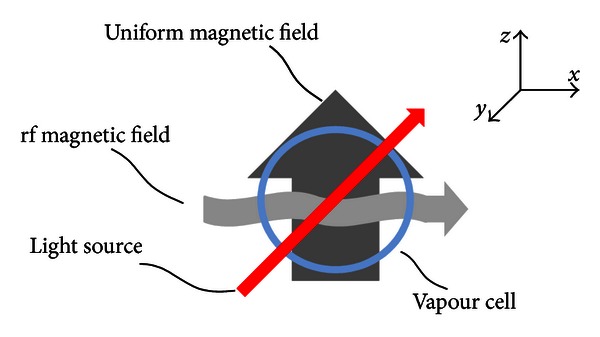
The generic configuration of an optical Mx magnetometer. The vapor cell contains alkali atoms such as Cs atoms.

The generic configuration of an optical Mx magnetometer is shown in Figure 1. The core of the system is a glass cell filled with the vapor of one of the alkali metals that have only one electron in their outermost shell. Generally, cesium is used because it possesses only one stable isotope, ^133^Cs, with a nuclear spin *I* = 7/2. 

**Figure 2:**
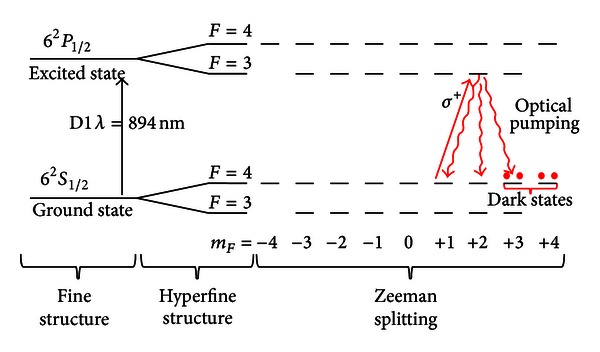
Fine, hyperfine structures and Zeeman splitting of the cesium D1 line with the optical pumping process and the dark states highlighted.

When a uniform dc external magnetic field is applied, each hyperfine level of cesium atoms splits into 2*F* + 1 Zeeman sublevels, as displayed in Figure 2. The light source is used both for pumping the Cs atoms to excited states and as a probe signal that senses an rf oscillating magnetic field modulating the absorption coefficient of the Cs atoms. Photons having a wavelength equal to the first absorption line of the alkali atoms are absorbed by the cesium atoms, thus moving them to the excited state. Figure 2 shows the optical pumping over the *F* = 4 → *F* = 3 transition of the cesium D1 absorption line using right circularly polarized light, which results in photon's angular momentum equal to +1. A Cs atom in the lower state with a quantum number *m*_*F*_ is allowed to move only to the upper level with *m*_*F*_′ = *m*_*F*_ + 1 because of total angular momentum conservation. A Cs atom that is in the excited state can decay via one of three possible decay channels and it spontaneously emits a photon that has equal probability to be sent in any direction, not necessary the direction of light beam. Therefore, there is a finite probability for the atom to decay into a level with a larger *m*_*F*_ value; thus, if the pumping process is repeated several times the atom moves to the two outermost states (*m*_*F*_ = 3 and 4) and, eventually, can no longer be optically pumped because there is not a corresponding state in the excited level available for transition [8]. Since these atoms do not absorb light, they are called dark states. 

The Cs atoms in the dark states precess around the magnetic field axis at the Larmor frequency that is proportional to the field magnitude: *w*_*L*_ = *γ*|*B*_0_|, where *γ* is the gyromagnetic constant that for cesium is 2*π* · 3.5 Hz/nT. During this time, the atom spin accumulates an increasing phase angle. 

The spin state of an atom can be changed to an absorbing (nondark) state through the absorption of resonant radio frequency radiation. A change in the spin orientation causes a Cs atom to come out of the dark state into an absorbing state. Amplitude modulation of the transmitted light can be achieved when a large fraction of the atoms precess coherently in phase [9]. To maintain this coherence and synchronize the precession of atomic spins, the rf driving magnetic field must be resonant with the Larmor frequency, that is, *w*_rf_ = *w*_*L*_. The ensemble average of the magnetic moments associated with the spins can be treated as a classical magnetization vector *M*. The precession of *M* around the total (dc and ac) magnetic field (*B*_tot_ = *zB*_0_ + *xB*_rf_cos⁡⁡ (*w*_rf_ *t*)) is given by the Bloch equations [10]:

(1) $$\begin{array}{c} \left(\begin{array}{c} {\dot{M}}_{x}\\ {\dot{M}}_{y}\\ {\dot{M}}_{z} \end{array}\right)=\left(\begin{array}{c} M_{x}\\ M_{y}\\ M_{z} \end{array}\right)\times \left(\begin{array}{c} \gamma B_{\text{rf}}\cos w_{\text{rf}}t\\ 0\\ \gamma B_{0} \end{array}\right)-\left(\begin{array}{c} \Gamma_{2}M_{x}\\ \Gamma_{2}M_{y}\\ \Gamma_{1}M_{z} \end{array}\right), \end{array}$$

where Γ_1_ and Γ_2_ are the longitudinal and the transverse relaxation time, respectively. The calculated in-phase and quadrature amplitudes and the phase shift of light power with respect to the driving rf magnetic field, *B*_rf_, are given by

(2) $$\begin{array}{c} P_{\text{ip}}\left(\delta \right)=-P_{0}\sin\left(2\theta \right)\frac{\Omega_{\text{rf}}\delta }{\Omega_{\text{rf}}^{2}\Gamma_{2}/\Gamma_{1}+\Gamma_{2}^{2}+\delta^{2}}; \end{array}$$

(3) $$\begin{array}{c} P_{\text{qu}}\left(\delta \right)=-P_{0}\sin\left(2\theta \right)\frac{\Omega_{\text{rf}}\Gamma_{2}}{\Omega_{\text{rf}}^{2}\Gamma_{2}/\Gamma_{1}+\Gamma_{2}^{2}+\delta^{2}}; \end{array}$$

(4) $$\begin{array}{c} \phi =\arctan\frac{\Gamma_{2}}{\delta }, \end{array}$$

where *Ω*_rf_ = *γ* · *w*_rf_ is the Rabi frequency, *δ* = *w*_rf_ − *w*_*L*_ is the frequency detuning from the Larmor frequency, and **θ** is the angle between the laser beam and *B*_0_.

Several important parameters can affect the magnetometer sensitivity, namely, the laser power; the laser beam profile, the rf field power, the cell size, the buffer-gas pressure, and the density of Cs atoms (which depends on the temperature). Typically, the magnetometer spatial resolution depends on the cell dimensions. In this paper, we focus the investigation on the effects of optical power intensity and vapor cell temperature variations on the sensitivity and bandwidth of the optical Mx magnetometer.

## 3. Experimental Setup

**Figure 3:**
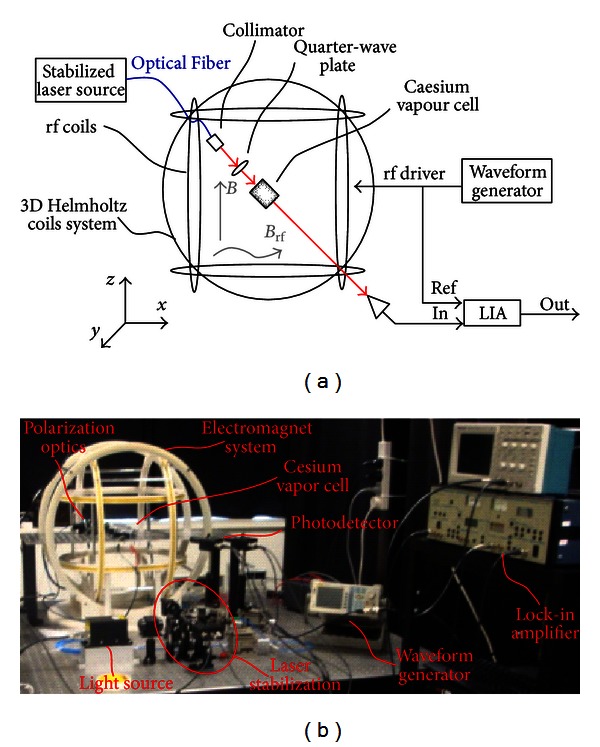
(a) Block diagram and (b) photo of the experimental setup for demonstrating the concept of the optical Mx magnetometer.

The Mx magnetometer used in the experiment is shown in Figures 3(a) and 3(b). The core of the instrument is a quartz-made cylindrical cell containing Cesium vapor. Also, Neon at 34 Torr and Argon at 6 Torr are added to the Cs vapor in order to reduce atom collisions. The cell diameter and length are 21 mm and 75 mm, respectively, yielding a spatial resolution of about 53 mm. In the experiments, the gas pressure inside the cell was increased by increasing the temperature using hot water flowing into a silicon pipe wrapped around the cell. The vapor cell was placed in the center of an electromagnet that generates a dc magnetic field in order to cancel the geomagnetic field and supply a uniform magnetization along the appropriate direction inside the vapor cell. The used electromagnet consists of two parts: (i) a 3D DC coils of dimension 580 mm × 530 mm × 640 mm providing a magnetic field with a uniformity better than 1% in the central region and (ii) an additional pair of coils that generate a small-magnitude rf magnetic field along the *x*-axis. Each coil pair of the electromagnet was independently driven by a digital power supply to cancel the geomagnetic field along the *x*- and *y*-directions and generate a uniform magnetic field along the *z* axis. The intensity of the magnetic field at the center of the electromagnet was 13 *μ*T, as measured by a three-axis smart digital magnetometer Honeywell HMR2300. The AC coils were driven by a waveform generator (Agilent, model 33250A) to produce an rf magnetic field of intensity 200 nT, oscillating at 45.5 kHz along the *x*-axis.

An external-cavity semiconductor laser (made by Uniquanta Technology, Beijing, China) was used as light source for both pumping and probing. The laser wavelength was tuned to 894 nm which is equal the cesium D1 absorption line *F* = 4 → *F* = 3 transition and stabilized using saturation spectroscopy in an auxiliary cell. The frequency stabilized light was coupled into a single-mode polarization maintaining optical fiber of 5 *μ*m core diameter and delivered to the vapor cell which was placed inside the electromagnet. At the cell location, the light was collimated, providing an output beam diameter of 1.6 mm, and right circularly polarized using a half-wave plate, an optical polarizer, and a quarter-wave plate. The laser beam was transmitted through the vapor cell at an angle of 45 degrees with respect to both the *z*- and the *x*-axes. The output light beam was focused into a photodiode (PED801 from UniQuanta Technology), which was placed outside the electromagnet in order to reduce the magnetic interference produced by the transimpedance amplifier of the photodiode package. A spectrum analyzer (Agilent, model E4407B) was used to measure the power spectral density of the photodiode output in order to calculate the signal-to-noise ratio and hence the intrinsic and actual sensitivity of the optical Mx magnetometer. Finally, a lock-in amplifier (Stanford Research Systems, model SR530) was used to measure the phase shift between the photocurrent detected by the photodiode with respect to the oscillating rf magnetic field, and the in-phase component and the quadrature component. Note that, by sweeping the frequency of the rf magnetic field along the Larmor frequency, the half width at half maximum (HWHM) of the in-phase, the quadrature and the phase signals could be measured.

It is important to mention that all the experimental results reported below were performed in laboratory environment without any magnetic shield. The uniform magnetic field along the *z*-axis was 13 *μ*T corresponding of a Larmor frequency of 45.5 kHz. It is also important to note that when the measurements were carried out with different uniform dc magnetic field intensities the performances of the magnetometer were not affected. The intensity of the rf magnetic field was 200 nT. In the experiments, the optical Mx magnetometer was operated in free-running mode without the use of a feedback loop between the lock-in amplifier and the signal generator to lock the rf frequency to the Larmor frequency. Particularly, the dependence of the sensitivity and the bandwidth of the optical Mx magnetometer on cell temperature and optical power was investigated for different input optical power levels over a cell temperature range of 23°C to 55°C.

## 4. Results and Discussion

### 4.1. Sensitivity

The sensitivity of the optical Mx magnetometer is defined as the smallest change in magnetic field that can be detected by the magnetometer. The sensitivity can be calculated using the Cramer-Rao equation [11]:

(5) $$\begin{array}{c} \rho =\frac{4\sqrt{3}\sqrt{f_{\text{bw}}}}{\gamma \text{SNR}}, \end{array}$$

where *f*_bw_ is the bandwidth, *γ* is the gyromagnetic constant and SNR is the signal-to-noise ratio of the magnetometer. All the following results refer to a 1 Hz bandwidth.

**Figure 4:**
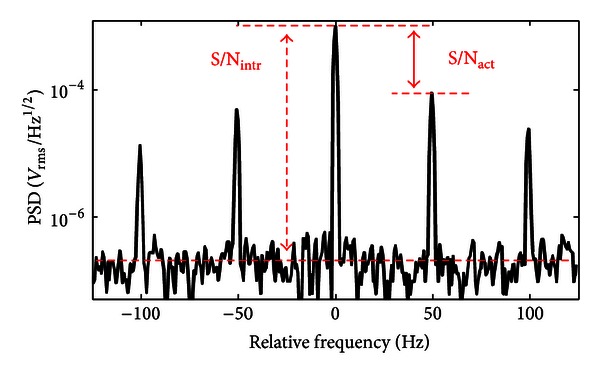
Power spectral density of the photodiode output normalized to the resonance frequency. The spectrum was measured in a 1 Hz bandwidth. The signal was recorded with optical power of 20 *μ*W and cell temperature of 50°C. The intrinsic and actual signal-to-noise ratios are highlighted.

The SNR can be calculated from the measured power spectral density (PSD) of the photodiode output, as illustrated in Figure 4. Since the magnetometer was operated in a magnetically-unshielded environment, all the measurements were dominated by the magnetic field noise. 

**Figure 5:**
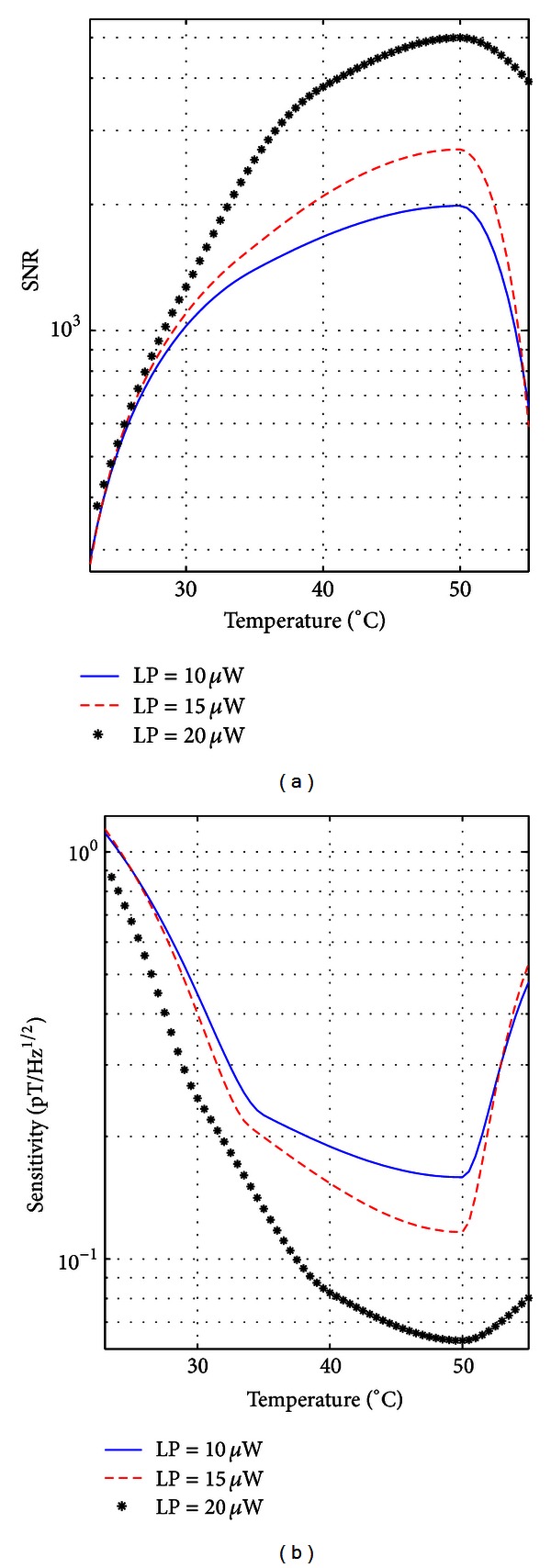
(a) Intrinsic signal-to-noise ratio (SNR) and (b) intrinsic sensitivity, measured in a 1 Hz bandwidth versus cell temperature for input optical power of 10 *μ*W, 15 *μ*W, and 20 *μ*W.

The intrinsic signal-to-noise ratio was calculated with the noise being the intrinsic rms noise of the magnetometer, measured over 100 Hz from the resonance frequency. The actual signal-to-noise ratio was calculated by taking into account the sidebands induced by the 50 Hz magnetic noise produced by power lines.  Figure 5 shows the intrinsic SNR (a) and the intrinsic sensitivity (b) of the magnetometer versus the cell temperature for different input optical power levels.

The intrinsic SNR, and hence the intrinsic sensitivity, curve exhibits a similar trend for all input optical power levels, namely, the SNR increases with increasing temperature, reaching a maximum value around 50°C before it starts to decrease. Moreover, increasing the input optical power increases the intrinsic SNR, and hence the intrinsic sensitivity. In fact, when the temperature increases, the gas pressure inside the vapor cell increases, resulting in a higher number of atoms interacting with the light and coherently precessing around the magnetic field at the Larmor frequency. Furthermore, increasing the input optical power results in a greater number of atoms being optically pumped. However, when the gas pressure is too high, the collisions of atoms with each other or with the walls of the cell lead to phase incoherence during precession, thus reducing the SNR performance of the magnetometer. The best performance of the magnetometer was obtained with an input optical power of 20 *μ*W and a cell temperature of 50°C. The measured intrinsic SNR was 5000 and the intrinsic sensitivity was 63 fT/Hz^1/2^, measured in a 1 Hz bandwidth. 

**Figure 6:**
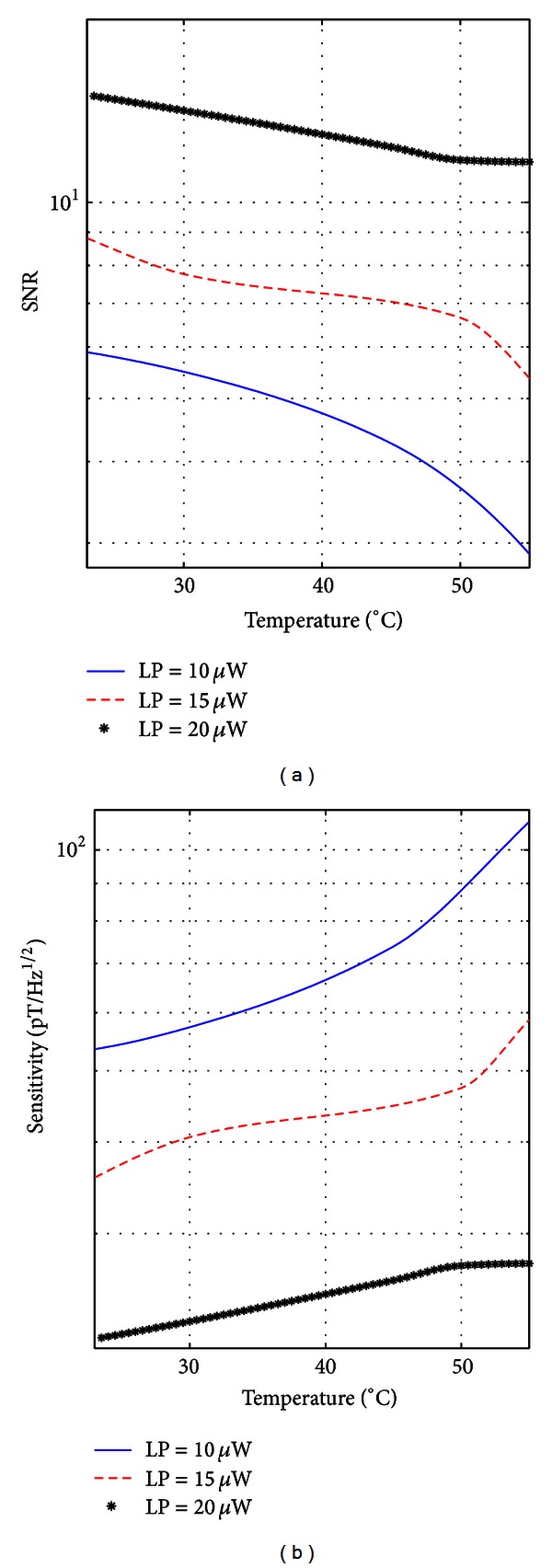
(a) Actual signal-to-noise ratio (SNR) and (b) actual sensitivity versus cell temperature, measured in a 1 Hz bandwidth for an input optical light power of 10 *μ*W, 15 *μ*W, and 20 *μ*W.

To obtain the actual SNR and the actual sensitivity of the magnetometer, the SNR was calculated by taking into account the external magnetic noise. Obviously, the actual sensitivity is strongly dependent on the location of the magnetometer. All the experiments reported in this paper were performed in the laboratory environment without any magnetic shield. This resulted in a very poor actual signal-to-noise ratio, and hence, a very low actual sensitivity. Figures 6(a) and 6(b) show the actual SNR and the actual sensitivity of the magnetometer, respectively. Also noticed in this case that the actual SNR increases (and hence the actual sensitivity is enhanced) with increasing the input optical power. However, the actual SNR decreases with increasing the cell temperature, mainly because of the higher phase incoherence that reduces the output signal level. With an input optical power of 20 *μ*W and a cell temperature of 50°C, the actual SNR dropped to 11.6 and the actual sensitivity was 27 pT/Hz^1/2^, measured in a 1 Hz bandwidth. The best actual sensitivity was obtained with an input optical power of 20 *μ*W at room temperature (23°C). The corresponding actual SNR was 14.5 and the actual sensitivity was 21 pT/Hz^1/2^, measured in a 1 Hz bandwidth.

### 4.2. Bandwidth

**Figure 7:**
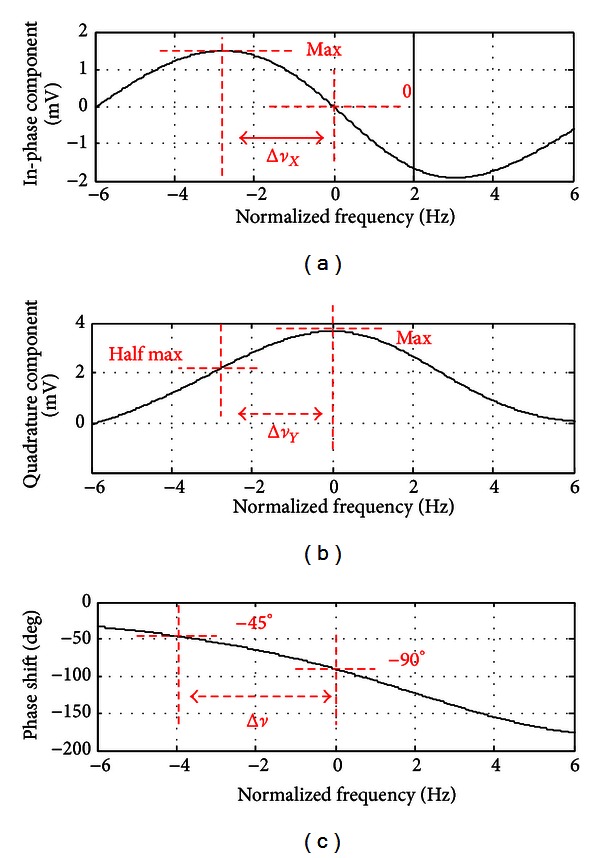
Measured in-phase component (a); quadrature component (b) and phase shift (c) between the photo diode output and the driving rf magnetic field. All signals were measured in a magnetically unshielded environment by continuously sweeping the driving rf frequency along the resonance frequency over a 6-second time range. It is important to note that the magnetically induced 50 Hz interference signal was suppressed by a notch filter. The input optical power was 20 *μ*W and the vapor cell temperature 48°C. The HWHM bandwidth is highlighted for each measured output signal.

Another important feature of a magnetometer is its bandwidth, that is, how fast the magnetometer responds to changes in the magnetic field. The bandwidth, *f*_bw_, of the magnetometer can be calculated as [12]:

(6) $$\begin{array}{c} f_{\text{bw}}=\frac{\pi }{2}\Delta \nu , \end{array}$$

where Δ*ν* is the half width at half maximum (HWHM) bandwidth of the phase signal measured in hertz, as illustrated in Figure 7(c). 

Figures 7(a) and 7(b) show, respectively, the in-phase and quadrature components of the lock-in amplifier output versus the normalized frequency, predicted by (2) and (3). Figure 7(c) shows the phase shift between the photodiode output and the driving rf magnetic field, *B*_rf_, predicted by (4). All signals in Figures 7(a)–7(c) were measured in a magnetically unshielded environment by continuously sweeping the driving rf frequency along the resonance frequency over a 6-second time range. It is important to note that the magnetically induced 50 Hz interference signal was suppressed by a notch filter. It is obvious from Figures 7(a)–7(c) that the HWHM bandwidths of both the in-phase signal, Δ*ν*_*X*_, and the quadrature signal, Δ*ν*_*Y*_, are equal and smaller than the HWHM bandwidth, Δ*ν*, of the phase-shift signal. This agrees with the theoretical prediction using (2)–(4). In fact, the phase shift is the only signal that is not dependent on the rf magnetic field. Therefore, for the accurate evaluation of the bandwidth of the magnetometer, *f*_bw_, it is important to use into (6) the HWHM bandwidth, Δ*ν*, measured from the phase shift rather than the HWHM bandwidths, Δ*ν*_*X*_ and Δ*ν*_*Y*_, measured from the in-phase and quadrature components, respectively. 

**Figure 8:**
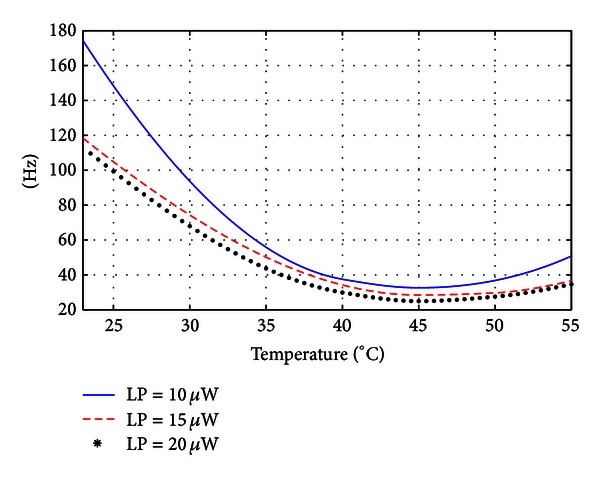
Intrinsic bandwidth versus cell temperature for an input optical light power of 10 *μ*W, 15 *μ*W, and 20 *μ*W.

Figure 8 shows the magnetometer bandwidth versus cell temperature for input optical power levels of 10 *μ*W, 15 *μ*W, and 20 *μ*W. The bandwidth exhibits a similar trend for all input optical power levels, initially decreasing with increasing the cell temperature until reaching its minimum around 45°C. Subsequently the bandwidth increases with increasing the cell temperature. The bandwidth depends on the transverse relaxation time Γ_2_, as evident from (4) and (6). An increase in the cell temperature increases the vapor pressure, which results in a larger number of atoms interacting with the light, leading to a longer relaxation time (smaller bandwidth). However, at high temperature, the number of collisions between atoms or with the walls of the cell increases significantly, leading to a shorter relaxation time and hence a larger bandwidth. This explains the existence of a critical temperature (around 45°) at which the intrinsic bandwidth is minimum. 

In the experiments, the measured maximum bandwidth was 175 Hz, obtained at room temperature (23°C) with an input optical power of 10 *μ*W (blue curve in Figure 8), while the minimum bandwidth was 25 Hz, obtained with an input optical power of 20 *μ*W at a temperature of 45°C (black curve in Figure 8).

### 4.3. Low-Amplitude Magnetic Field Measurement

The ultimate intrinsic sensitivity of the magnetometer can be calculated using (5). The best performance of the magnetometer was obtained for an input optical power of 20 *μ*W at cell temperature of 48°C; the ultimate intrinsic sensitivity was 327 fT/Hz^1/2^ over a bandwidth of 26 Hz. However, the external magnetic noise generated by power lines and surrounding equipment caused the actual ultimate sensitivity of the magnetometer to drop to 130 pT/Hz^1/2^ over a bandwidth of 26 Hz. 

The magnetometer in its optimal configuration (input optical power of 20 *μ*W at cell temperature of 48°C) was then used to measure an applied small-signal sinusoidal magnetic field of amplitude 15 pT oscillating at 25 Hz, which was generated by a test coil placed at a distance of 6 cm from the vapor cell. For this measurement, the uniform dc magnetic field was 13 *μ*T, corresponding to a Larmor frequency of 45.5 kHz. The frequency of the rf magnetic field was then set at 45.5 kHz resulting in a phase shift of −90 degrees between the photodiode output and the driving rf signal, as predicted by (4), and verified experimentally by the result shown in Figure 7(c). When another 25 Hz small-amplitude magnetic field was applied in addition to the dc and rf magnetic fields, the Larmor frequency changed and no more resonance occurred, causing the phase shift between the photodiode output and the driving rf signal to oscillate around −90 degrees at 25 Hz. This enabled the measurement of the new Larmor frequency and hence the calculation of the magnitude of the 25 Hz small-amplitude magnetic field, which is proportional to the new Larmor frequency. 

**Figure 9:**
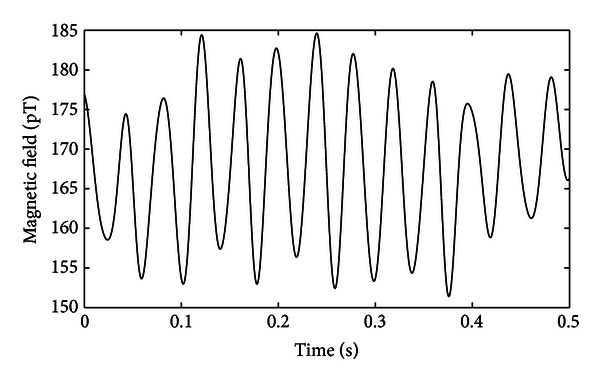
Measured 15 pT peak oscillating field at frequency of 25 Hz filtered with a low-pass filter with cutoff frequency of 50 Hz.

Figure 9 shows the magnetic field calculated from the measurement of the Larmor frequency after a 25 Hz 15 pT magnetic field was applied. A lowpass filter of a cutoff frequency of 50 Hz was used after the lock-in amplifier in order to remove the power line noise (at 50 Hz and its harmonics) as well as all high frequencies noise. It is obvious from Figure 9 that the small-signal 25 Hz magnetic field could be recovered (peak-to-peak magnitude is around 33 pT), demonstrating the capability of the optical Mx magnetometer to measure ultra-low-amplitude magnetic fields.

## 5. Conclusion

An optically pumped quantum magnetometer on Mx configuration has been developed and its capability to measure ultra-low-amplitude magnetic fields has been experimentally demonstrated. A high intrinsic sensitivity of 63 fT/Hz^1/2^ measured in a 1 Hz bandwidth has been achieved with an input optical power of 20 *μ*W at a vapor cell temperature of 50°C. Experimental results have shown that the environmental noise can significantly drop the magnetometer sensitivity by several orders of magnitude to as low as 27 pT/Hz^1/2^. A high actual sensitivity of 21 pT/Hz^1/2^ has been attained with an input optical power of 20 *μ*W at room temperature. The measured bandwidth of the magnetometer has been shown to vary between 100 Hz at room temperature and 25 Hz at 45°C. Experimental results have also shown that the ultimate best intrinsic sensitivity (327 fT/Hz^1/2^) calculated over the measured bandwidth (26 Hz) can be attained with an input optical power of 20 *μ*W at a vapor cell temperature of 48°C and that the environmental noise reduces this sensitivity to 130 pT/Hz^1/2^. Finally, the ability of the magnetometer to detect a 25 Hz sinusoidal magnetic field of amplitude as low as 15 pT has experimentally been demonstrated.
